# Formation of Fenestrae in Murine Liver Sinusoids Depends on Plasmalemma Vesicle-Associated Protein and Is Required for Lipoprotein Passage

**DOI:** 10.1371/journal.pone.0115005

**Published:** 2014-12-26

**Authors:** Leonie Herrnberger, Robert Hennig, Werner Kremer, Claus Hellerbrand, Achim Goepferich, Hans Robert Kalbitzer, Ernst R. Tamm

**Affiliations:** 1 Department of Human Anatomy and Embryology, University of Regensburg, Regensburg, Germany; 2 Department of Pharmaceutical Technology, University of Regensburg, Regensburg, Germany; 3 Department of Biophysics and Physical Biochemistry, and Centre of Magnetic Resonance in Chemistry and Biomedicine, University of Regensburg, Regensburg, Germany; 4 Department of Internal Medicine I, University Hospital Regensburg, Regensburg, Germany; Centro de Investigación en Medicina Aplicada (CIMA), Spain

## Abstract

Liver sinusoidal endothelial cells (LSEC) are characterized by the presence of fenestrations that are not bridged by a diaphragm. The molecular mechanisms that control the formation of the fenestrations are largely unclear. Here we report that mice, which are deficient in plasmalemma vesicle-associated protein (PLVAP), develop a distinct phenotype that is caused by the lack of sinusoidal fenestrations. Fenestrations with a diaphragm were not observed in mouse LSEC at three weeks of age, but were present during embryonic life starting from embryonic day 12.5. PLVAP was expressed in LSEC of wild-type mice, but not in that of *Plvap*-deficient littermates. *Plvap^-/-^* LSEC showed a pronounced and highly significant reduction in the number of fenestrations, a finding, which was seen both by transmission and scanning electron microscopy. The lack of fenestrations was associated with an impaired passage of macromolecules such as FITC-dextran and quantum dot nanoparticles from the sinusoidal lumen into Disse's space. *Plvap*-deficient mice suffered from a pronounced hyperlipoproteinemia as evidenced by milky plasma and the presence of lipid granules that occluded kidney and liver capillaries. By NMR spectroscopy of plasma, the nature of hyperlipoproteinemia was identified as massive accumulation of chylomicron remnants. Plasma levels of low density lipoproteins (LDL) were also significantly increased as were those of cholesterol and triglycerides. In contrast, plasma levels of high density lipoproteins (HDL), albumin and total protein were reduced. At around three weeks of life, *Plvap*-deficient livers developed extensive multivesicular steatosis, steatohepatitis, and fibrosis. PLVAP is critically required for the formation of fenestrations in LSEC. Lack of fenestrations caused by PLVAP deficiency substantially impairs the passage of chylomicron remnants between liver sinusoids and hepatocytes, and finally leads to liver damage.

## Introduction

Endothelial cells of liver sinusoids differ from that of other capillaries because of the presence of open fenestrations and the absence of a basal lamina [1]–[4]. The diameter of the sinusoidal fenestrations varies between different species and measures 107±1.5 nm (mean ± SEM) in the human liver [5], while for the mouse liver an average diameter of 141±5.4 nm (mean ± SEM) [5] was determined on tangentially sectioned transmission electron micrographs, a method that largely avoids shrinkage effects [6]. A specific function of liver sinusoids is the filtration of particles that are exchanged between the sinusoidal lumen and the space of Disse, allowing only particles smaller than the fenestrae to reach the hepatocytes or to leave the space of Disse [7]. Such a filter function appears to be especially important for the passage of lipoproteins. In support of this are observations of numerous chylomicron remnants with a size smaller than the diameter of fenestrations in the space of Disse of breastfeeded rat pups [8]. Moreover, several studies found evidence for a negative correlation between fenestration size and number, and the plasma concentration of larger lipoproteins [1], [9]. Still, others did not find evidence supporting the assumption that the diameter of liver sinusoidal fenestrae is a major determinant of plasma lipoprotein levels [10].

The proliferation and survival of sinusoidal endothelial cells [11], as well as their capability to form fenestrations [9], depend on vascular endothelial growth factor (VEGF) that is secreted from hepatocytes [9]. Otherwise very little is known about the signaling mechanisms that modulate the maintenance and turnover of fenestrations in liver sinusoids. Multiple agents were shown to modulate size and numbers of fenestrations [1]. Still, a specific molecule that is part of the biological process behind pore formation in the wall of a sinusoidal endothelial cell in the liver has not been identified.

Capillary endothelial cells of other organs with high demands for the passages of fluids and solutes differ from sinusoidal endothelial cells of the liver as they form fenestrae that are covered by a thin 5–6 nm non-membranous diaphragm [3], [12]. The only protein that has been identified to be a component of endothelial diaphragms is plasmalemma vesicle-associated protein (PLVAP, synonyms: MECA32, PV-1), an endothelial-specific, cationic, integral membrane glycoprotein [13], [14]. We and others recently showed that PLVAP is critically required for the formation of endothelial diaphragms which are completely absent in endothelia of *Plvap*-deficient mice [15], [16].

Here we report that the absence of PLVAP in *Plvap*-deficient mice causes a pronounced reduction in the number of fenestrations in sinusoidal endothelial cells of the liver. Our results indicate that PLVAP is not only required for the formation of endothelial diaphragms, but also for the formation of endothelial fenestrae, even of those without diaphragms. The lack of sinusoidal fenestrations impairs the passage of macromolecules between the sinusoidal lumen and Disse's space. A direct result is the presence of a severe hyperlipoproteinemia caused by the massive accumulation of chylomicron remnants in the plasma of *Plvap*-deficient animals. The phenotype provides clear molecular evidence for the assumption that open fenestrae in liver sinusoidal cells are critically required for the passage of lipoproteins.

## Materials and Methods

### Animals

All procedures conformed to the tenets of the National Institutes of Health Guidelines on the Care and Use of Animals in Research, the EU Directive 2010/63/E, institutional guidelines and were approved by the local authority (Regierung der Oberpfalz, Bavaria, Germany) and the Animal Care and Use Committee (Tierschutzausschuss) of the University of Regensburg. Mice were housed under standard laboratory conditions (12–12 h light-dark cycle, lights on at 6 am, 22°C, and 60% humidity) with food and water *ad libitum*. *Plvap^-/-^* mice were used as described previously [17]. Mice were kept in a mixed C57BL/6N/FVB-N background. All experiments were performed in mice of either sex. Genotyping was routinely performed by PCR analysis, using two upper primers located in intron 1 of *Plvap* (5′-AGAGCCTTCTCTGCCAAGTG-3′) and in the inserted cassette (5′-TCTCATGCTGGACTTCTTCG-3′) and a lower primer located in intron 1 downstream of the cassette (5′-GGCTAGCCTGAGCTACAGAGG-3′) resulting in a 672 bp PCR fragment for the wild-type allele and a 552 bp fragment for the targeted allele. DNA was obtained from tail tips. PCR was performed in 15 µl reaction volumes containing standard buffer, 0.1 µM of each primer, 1 mM dNTPs, 2.5 mM MgCl_2_, 8% glycerol, 0.2 mM cresol red sodium salt (Sigma, Taufkirchen, Germany) and 0.25 U *Taq* polymerase (New England Biolabs, Taufkirchen, Germany). The cycling conditions consisted of an initial 3 min denaturing step at 94°C, followed by 34 cycles for 30 s at 94°C, for 1 min at 65°C, and for 1 min 15 s at 72°C.

### Western blot analysis

Fresh lung samples were homogenized in RIPA buffer (150 mM NaCl, 1% NP-40, 0.5% deoxycholic acid, 0.1% SDS, and 50 mM Tris), and insoluble constituents were removed by centrifugation. For western blot analysis of PLVAP up to 30 µg of protein were subjected to 10% SDS-PAGE and transferred onto a PVDF membrane (Roche Diagnostics GmbH, Mannheim, Germany) by semidry blotting. After blocking with 5% BSA in TBS-T, membranes were incubated overnight with rat anti-pan ECA (Meca32) IgG_2a_ antibodies (Santa Cruz Biotechnology, Santa Cruz, CA, USA), diluted 1∶250 in 0.5% BSA in TBS-T. After washing in TBS-T, membranes were hybridized with HRP-conjugated chicken anti-rat antibodies (Santa Cruz), diluted 1∶1000 in 0.5% BSA in TBS-T. For visualization, membranes were incubated in Luminata Forte Western HRP substrate (Millipore Corporation, Billerica, MA, USA) and visualized on a LAS 3000 Imager work station (Fujiifilm, Düsseldorf, Germany). As loading control, membranes were incubated with HRP-conjugated rabbit anti-GAPDH antibodies (Cell Signaling, Technologies, Beverly, MA), diluted 1∶1000 in 0.5% BSA in TBS-T.

### RNA analysis

Total RNA from fresh lung and liver samples was extracted with peqGold Trifast (Peqlab, Erlangen, Germany) according to manufacturer's instructions. First-strand cDNA from total RNA was generated using qScript cDNA synthesis kit (Quanta BioScience Inc., Gaithersburg, MD, USA) according to the manufacturer's recommendations. RNA that was not reverse transcribed into cDNA served as negative control for real-time RT-PCR and RT-PCR. Primer pairs (Table 1) were purchased from Invitrogen (Darmstadt, Germany) and were designed to extend over exon–intron boundaries. Real-time RT-PCR was performed on a BioRad iQ5 real-time RT-PCR detection system (BioRad) with the temperature profile as follows: 40 cycles of 10 s melting at 95°C and 40 s of annealing and extension at 60°C. In initial experiments, the potential housekeeping genes for real-time RT-PCR were identified for each tissue and best results were obtained for GAPDH. Quantification was performed using BioRad iQ5 standard edition (version 2.0.148.60623) software (BioRad). For RT-PCR, first strand cDNA was generated using qScript cDNA synthesis kit (Quanta BioScience Inc., Gaithersburg, MD, USA) according to the manufacturer's recommendations. RNA that was not reverse transcribed into cDNA served as negative control. RT-PCR was performed in 25 µl reaction volumes containing standard buffer, 0.1 µM of each primer, 1 mM dNTPs, 2.5 mM MgCl_2_, and 0.25 U *Taq* polymerase (New England Biolabs, Taufkirchen, Germany). The cycling conditions consisted of an initial 3 min denaturing step at 94°C, followed by 37 cycles for 30 s at 94°C, for 50 s at 63°C, and for 90 s at 72°C.

**Table:** **Table 1.** Primers used for real-time RT-PCR and RT-PCR.

**Real-time RT-PCR**	
mPlvap fwd	5'-TCAACAAGACCTGCGAAGC-3'
mPlvap rev	5'-AGCACACTGCCTTCTCCTTG-3'
mGAPDH fwd	5'-TGTCCGTCGTGGATCTGAC-3'
mGAPDH rev	5'-CCTGCTTCACCACCTTCTTG-3'
**RT-PCR**	
mPlvap fwd	5'-CTATCATCCTGAGCGAGAAGC-3'
mPlvap rev	5'-GCAGCAGGGTTGACTACAGG-3'

### Electron microscopy

For transmission electron microscopy of mouse livers, deeply anesthetized mice (120 mg/kg body weight i.m. ketamine and 8 mg/kg body weight i.m. xylazine) were either fixed by intracardial perfusion with 2.5% glutaraldehyde in 0.1 M cacodylate buffer (pH 7.4) followed by immersion fixation in 2.5% glutaraldehyde in 0.1 M cacodylate buffer (pH 7.4) overnight, or by immersion fixation alone. Afterwards, livers were treated with 1% OsO_4_, 0.8% K_4_[Fe(CN)_6_] in 0.1 M cacodylate buffer for 1.5 h, dehydrated with graded ethanol solutions and embedded in Epon (Roth, Karlsruhe, Germany). Semi-thin sections were stained with Richardson's stain. Ultrathin sections were stained with uranyl acetate and lead citrate, and analyzed on a transmission electron microscope (Libra, Zeiss). For scanning electron microscopy of mouse livers, deeply anesthetized mice (120 mg/kg body weight i.m. ketamine and 8 mg/kg of body weight i.m. xylazine) were fixed by intracardial perfusion with 2.5% glutaraldehyde in 0.1 M cacodylate buffer (pH 7.4). Liver samples were isolated and further fixed by immersion in 2.5% glutaraldehyde in 0.1 M cacodylate buffer (pH 7.4) overnight. After treatment with 1% OsO_4_, 0.8% K_4_[Fe(CN)_6_] in 0.1 M cacodylate buffer for 1.5 h, the samples were rinsed in four changes of 0.1 M cacodylate buffer and immersed in 30% N,N-dimethylformamide (DMF) dissolved in 0.1 M cacodylate buffer for 30 min. The liver samples were frozen with liquid nitrogen and were crushed into randomized pieces which were placed again in 30% DMF for 30 min, then rinsed twice with buffer and three changes of distilled water. The specimens were dehydrated in ascending acetone solutions and critical point dried using a CP drying apparatus E 3000 (Quorum Technologies Ltd, East Sussex, United Kingdom) with CO_2_ substitution. Dried samples were sputter-coated with platinum and examined with a field-emission scanning electron microscope (LEO 1530, Zeiss).

### Perfusion studies

For liver perfusion via the portal vein, mice were deeply anesthetized (120 mg/kg of body weight i.m. ketamine and 8 mg/kg body weight i.m. xylazine). The abdomen was opened and the portal vein was cannulated using a 27 gauge needle (Henry Schein, Melville, NY, USA). The needle was attached via disposable Mallinckrodt pressure tubing with an inner diameter of 0.050" and 24" length (Tyco Healthcare, Pleasanton, CA, USA) to a 3-way-stopcock (Fresenius Kabi AG, Bad Homburg, Germany). Two additional pressure tubes which are connected to two 10 ml syringes (Henry Schein, Melville, NY, USA) were attached to the 3-way stopcock. For perfusion, an infusion pump (PHD 2000, Harvard Apparatus, Holliston, MA, USA) was used. Fluorescein isothiocyanate-dextran (FITC-dextran, FD70, Sigma, Taufkirchen, Germany) was dissolved at a concentration of 50 mg/ml in PBS. Qdot 655 ITK Amino (PEG) quantum dots (Life Technologies GmbH, Darmstadt, Germany) were perfused at a total amount of 20 pmol for each liver, with an infusion rate of 1 ml/min, and an infusion volume of 1 ml. After start of the perfusion, the inferior vena cava was cut open. To fix the tissue after perfusion, the 3-way-stopcock was switched to the syringe filled with 4% PFA and perfusion was restarted with an infusion rate of 1 ml/min and an infusion volume of 1 ml. Finally, right liver lobes were collected and further processed for histology and immunohistochemistry.

### Light microscopy

Embryos were obtained from timed matings with noon of the day of vaginal plug discovery designated as 0.5 days of gestation (E0.5). Embryos and tissues were collected and fixed by immersion in 4% PFA for 4 h. After fixation, embryos and tissue samples were washed in 0.1 M phosphate buffer (pH7.4), processed through several graded alcohol and xylenes, and embedded in paraffin. For frozen sections, the embryos and tissues were equilibrated in 10, 20, and 30% sucrose for 4 h and embedded in Tissue Tek optimal cooling temperature (OCT) compound (Sakura Finetek Europe B.V., Zoeterwoude, NL) and cooled at −20°C. After removal of paraffin, the sections were stained with hematoxylin and eosin (H&E) and analyzed using a Zeiss Axio Imager microscope (Carl Zeiss AG, Oberkochen, Germany). For sirius red staining, de-waxed sections were stained with Weigert's hematoxylin (1% hematoxylin, pH 5.0–7.2 in 96% isopropyl alcohol) for 8 min and then washed for 10 min in running tap water. Afterwards the sections were stained with 0.1% picro-sirius in saturated aqueous solution (Waldeck GmbH & Co., Division Chroma, Münster, Germany) for 1 h. After two washing steps in acidified water (0.5% acetic acid diluted in distilled water), the water was physically removed from the slides and the sections were dehydrated through three steps of 100% ethanol for 10 min. Afterwards the sections were cleared in two steps of 100% xylenes for 10 min, mounted with DePeX (Serva Feinbiochemica, Heidelberg, Germany) and examined using a Zeiss Axio Imager microscope. For staining for periodic acid-Schiff (PAS) de-waxed paraffin sections were oxidized by 1% periodic acid dissolved in 70% ethanol for 10 min and washed 3 times in distilled water for 1 min. Afterwards, the aldehyde groups were detected by Schiff reagent and counterstained with Weigert's hematoxylin. Sections were processed through several graded alcohol and xylenes and mounted with DePeX. For staining of β-galactosidase activity, embryos were fixed in 2% glutaraldehyde in 5 mM EGTA (pH 7.3) and 2 mM MgCl_2_ dissolved in PBS for 4 h on ice with shaking. After three 30 min rinses in washing buffer (2 mM MgCl_2_, 0.01% sodium deoxycholate, 0.02% Nonidet-P40 in PBS), β-galactosidase activity was visualized in X-Gal staining solution (2 mM MgCl_2_, 0.01% sodium deoxycholate, 0.02% Nonidet-P40, 5 mM potassium ferrocyanide, 5 mM potassium ferricyanide, 1 mg/ml X-Gal in PBS). Tissues were stained for 24 h at 37°C in the dark, washed three times for 5 min in washing buffer, and embedded in Tissue-Tek for frozen sections. P-phenylendiamine (PPD, Carl Roth, Karlsruhe, Germany) staining was performed on semi-thin sections of liver and kidney. Sections were stained with 1% PPD solution (10 mg/ml PPD in 98% ethanol) for 30 min at room temperature in the dark. Afterwards the sections were differentiated with 100% ethanol and counterstained with methylene blue.

### Immunohistochemistry

Tissue samples were fixed by immersion in 4% PFA or Carnoy's fixative (60% methanol, 30% chloroform and 10% acetic acid) for 4 h, washed in 0.1 M phosphate buffer, and embedded in either paraffin or Tissue-Tek. For detection of PLVAP, CD31, and α-smooth muscle-actin, sections were blocked with 1% BSA, 0.2% cold water fish gelatin (Aurion, Wageningen, Netherlands), and 0.1% Triton-X (all in 0.1 M phosphate buffer) for 1 h at room temperature. After blocking, the sections were incubated with rat monoclonal antibodies pan ECA (clone MECA-32, IgG2a, 1∶50, Santa Cruz) specific against PLVAP [18], [19], goat anti-CD31/PECAM-1 IgG (1∶20, R&D systems, Wiesbaden, Germany) or goat anti-α-smooth muscle-actin IgG (α-SMA) (1∶200; GeneTex, Irvine, CA, USA). Donkey anti-rat IgG (1∶2500) or donkey anti-goat IgG (1∶2500) each conjugated to Cy3 (Jackson ImmunoResearch), or rabbit anti-goat IgG (1∶1000) conjugated to Alexa Fluor 488 (Life Technologies GmbH, Darmstadt, Germany) were used as secondary antibodies. For fibronectin staining, sections were blocked with 2% BSA and 0.1 M Triton-X in 0.1 M phosphate buffer for 1 h. After blocking, sections were incubated with rabbit anti-fibronectin (1∶500, Dako, Hamburg, Germany). Goat anti-rabbit IgG (1∶1000) conjugated to Alexa 488 (Life Technologies GmbH, Darmstadt, Germany) were used as secondary antibodies. For IBA-1 staining sections were pretreated with 0.05 M ammonium chloride for 30 min and with 0.5% Triton-X for 5 min. Afterwards, sections were blocked with 1% BSA, 0.2% cold water fish gelatin (Aurion, Wageningen, Netherlands), and 0.1% Triton-X (all in 0.1 M phosphate buffer) for 1 h at room temperature. After blocking, sections were incubated with rabbit anti IBA-1 (1∶100, Wako, Neuss, Germany). Goat anti-rabbit IgG (1∶1000) conjugated to Alexa 488 (Life Technologies GmbH, Darmstadt, Germany) were used as secondary antibodies. Primary antibodies were always incubated overnight at 4°C and secondary antibodies were incubated for 1 h at room temperature in the dark. Slides were mounted with a medium containing 4',6-diamidino-2-phenylindole (DAPI, Vectashield; Vector Laboratories, Burlington, CA, USA) and analyzed under a fluorescence microscope (Zeiss, Axio Imager). For negative controls, primary antibodies were omitted and the sections were incubated with secondary antibodies only.

### TUNEL staining

Apoptotic liver cells were detected by terminal deoxynucleotidyl transferase-mediated dUTP nick end labeling using the Deadend Fluorometric TUNEL system (Promega, Mannheim, Germany). Tissues were fixed in 4% PFA for 4 h. After removal of paraffin, TUNEL staining was performed according to the manufacturer's instructions. Slides were analyzed under a fluorescence microscope (Zeiss, Axio Imager).

### Plasma analysis

For collection of blood plasma for lipoprotein analysis, mice were euthanized with isoflurane and rapidly killed by decapitation to collect trunk blood. Blood samples were centrifuged at 4000 g for 15 min and plasma was stored at - 20°C. Hematocrit was evaluated by collecting trunk blood which was separated by standard laboratory methods using heparinized micro hematocrit tubes (Brand GmbH, Wertheim, Germany). Serum aspartate transaminase (AST), alanine transaminase (ALT), albumin, HDL, LDL and total protein levels were measured applying standard clinical laboratory tests. Total cholesterol and total triglycerides were measured by standard enzymatic tests performed on a Dimension Vista System (Siemens Healthcare Diagnostics GmbH, Eschborn, Germany).

### NMR spectroscopy

NMR spectra were recorded on Avance 800 and 600 NMR spectrometers (Bruker Biospin Karlsruhe, Germany) operating at 800.2 and 600.0 MHz proton resonance frequencies, respectively. Both spectrometers were equipped with a cryoprobe for obtaining a higher sensitivity. Spectra were recorded from blood plasma of individual mice at 310 K. If the sample volume was smaller than 500 µL, an appropriate amount of physiological extracellular buffer [20] was added up to 500 µL. Quantification of the plasma lipoproteins was obtained from NOESY-type 1D spectra [21] and a set of Oneshot [22] diffusion weighted ^1^H NMR spectra. The concentrations of the different lipoprotein subclasses defined by the hydrodynamic radii r_H_ was calculated according to methods published previously [23], [24]. Chemical shifts δ and hydrodynamic radii r_H_ were referenced to the methyl signal of lactate used as internal standard. The chemical shift of lactate was set to 1.365 ppm, since it is known that the usual reference compound DSS interacts with the lipoproteins. The hydrodynamic radius of Tris-buffer was experimentally determined as 0.307 nm in a separate set of experiments and used for the calibration of the hydrodynamic radii of the triplet signal in the lipoproteins. The obtained r_H_-values were associated with different types of lipoproteins from the data published for human lipoprotein subclasses [23]. The relative concentration changes of the different lipoproteins were calculated for 3 samples each of wild-type and *Plvap^-/-^* littermates.

### Quantitative analysis

For quantitative analysis of the number of fenestrations in liver sinusoids, the total number of fenestrations was counted along the endothelium comprising a total length of 2.5 mm in both wild-type and *Plvap^-/-^* littermates. The number of fenestrations per µm capillary length was determined and expressed as mean ± SEM. Statistical analysis was done by a two-tailed Student's t-test and *p* values ≤0.05 were considered to be statistically significant.

## Results

### The loss of PLVAP in sinusoidal endothelial cells is associated with a lack of fenestrations

**Figure 1:**
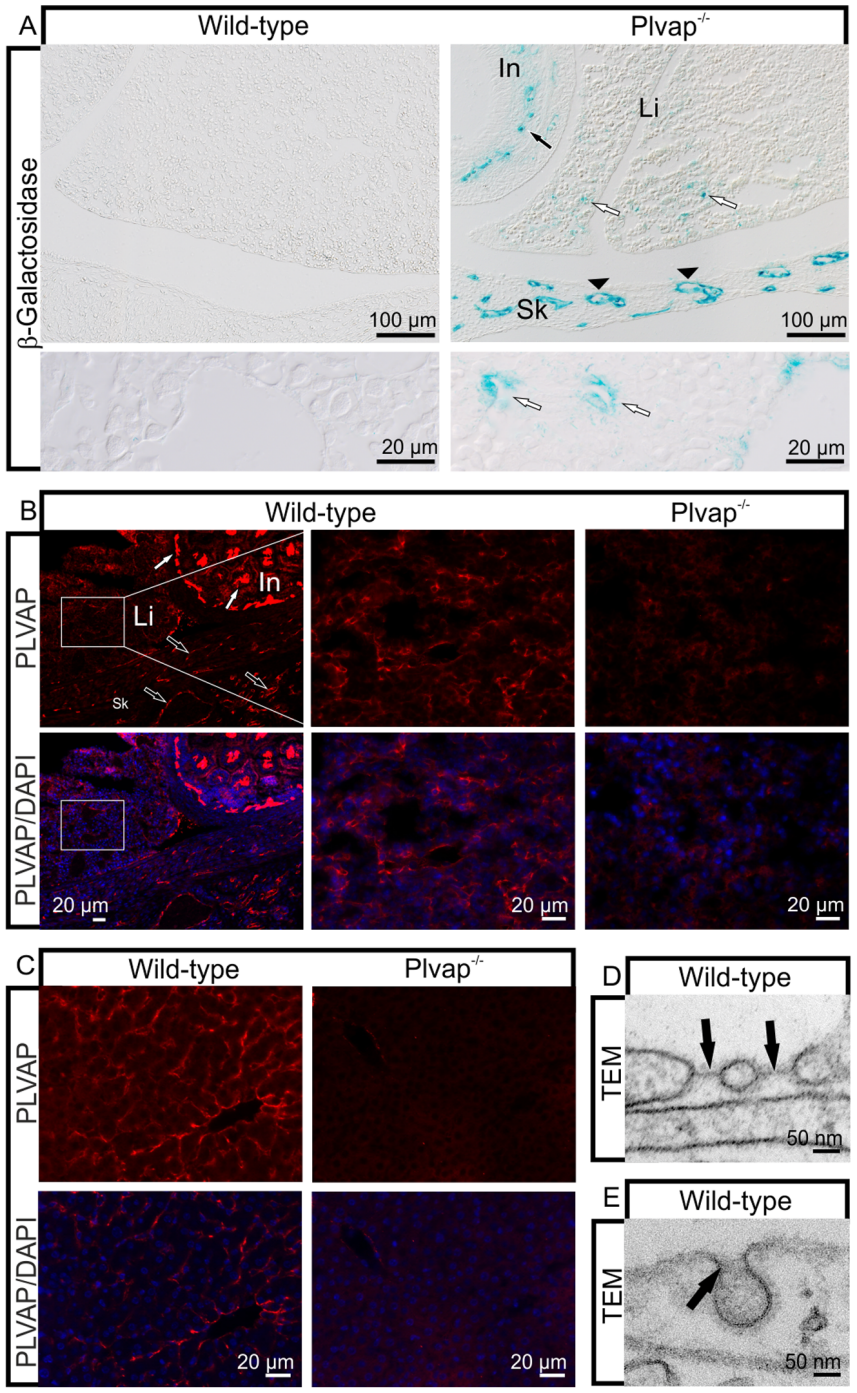
PLVAP in the liver. **A**, β-galactosidase stained sections through the livers of a *Plvap* ^-/-^ animal and its wild-type littermate at E15.5. Staining is seen in the sinusoidal endothelium of the *Plvap*-deficient embryo (white arrows), but not in the wild-type. Staining in the liver is patchy and weaker than the homogenous staining of capillaries in the small intestine (In, black arrows) or skin (Sk, arrowheads). Lower panels show magnification of the staining in liver sinusoids (white arrows). **B**, Immunolabeling for PLVAP (red) shows staining in the sinusoidal endothelium of a wild-type embryo at E16.5 (boxed areas in the left panel). Stronger immunostaining for PLVAP is seen in capillaries of the small intestine (white arrow) and the pancreas (black arrow). No staining is seen in the *Plvap*-deficient littermate (right panel). Middle panel shows magnification of the boxed panel. **C**, Liver staining for PLVAP (red) at P11 shows positive immunoreactivity in wild-type sinusoidal endothelium, but not in the *Plvap* ^-/-^ littermate. Nuclear DNA is labeled with DAPI (blue). **D** and **E**, TEM. Fenestrations bridged by 5 - 7 nm thick diaphragms (black arrows) are seen in wild-type sinusoidal endothelium at E12.5 (**D**). Caveolae of wild-type sinusoidal endothelium at P20 are not bridged by stomatal diaphragms (black arrow, **E**).

To analyze the expression of *Plvap* in the developing liver, we stained for β-galactosidase to detect the expression of the reporter gene *lacZ*. At embryonic day (E) 15.5, a time when fenestrations have been formed in the sinusoids of the mouse liver, we detected a positive staining in the sinusoidal endothelial cells of *Plvap*-deficient embryos (Fig. 1A). In contrast, staining was essentially absent in wild-type littermates (Fig. 1A). The β-galactosidase staining in the liver sinusoidal endothelium was patchy and considerably weaker than the more homogenous staining in the capillaries of the immediately adjacent small intestine or skin (Fig. 1A) which show strong *Plvap* expression [16]. Comparable results were obtained when localization of PLVAP was investigated by immunohistochemistry. At E16.5, liver sinusoidal cells of wild-type embryos showed a distinct immunoreactivity for PLVAP, a finding that was not observed in *Plvap* ^-/-^ embryos (Fig. 1B). The immunoreactivity was patchy and weaker than in the capillaries of the adjacent small intestine or pancreas (Fig. 1B). The immunoreactivity for PLVAP in the sinusoidal endothelial cells of wild-type animals at P11 was of similar intensity than in embryos, but more homogenous (Fig. 1C). Again, no immunoreactivity for PLVAP was observed in *Plvap* ^-/-^ littermates (Fig. 1C).

PLVAP is a component of the diaphragms covering endothelial fenestrae, transendothelial channels, and caveolae [14], [25]. Since the formation of fenestrae with diaphragms precedes that of open fenestrae during development of the sinusoidal endothelium in rat embryos [26], we assumed that the PLVAP staining in the mouse embryos was associated with the formation of diaphragmed fenestrae. Indeed, we observed fenestrae with a diaphragm in the sinusoidal endothelium of mouse embryos starting from E12.5 (Fig. 1D). As expected, fenestrae of sinusoidal endothelial cells in three-week-old mice were open while fenestrae or transendothelial channels covered by diaphragms were not observed. Also caveolae, which were only rarely observed in sinusoidal endothelial cells of wild-type pups after birth, did not form stomatal diaphragms (Fig 1E), a finding that corroborated results from a previous report [27]. Overall, our data did not support the concept that the presence of PLVAP in sinusoidal endothelial cells of the postnatal mouse liver is associated with the presence of endothelial diaphragms.

**Figure 2:**
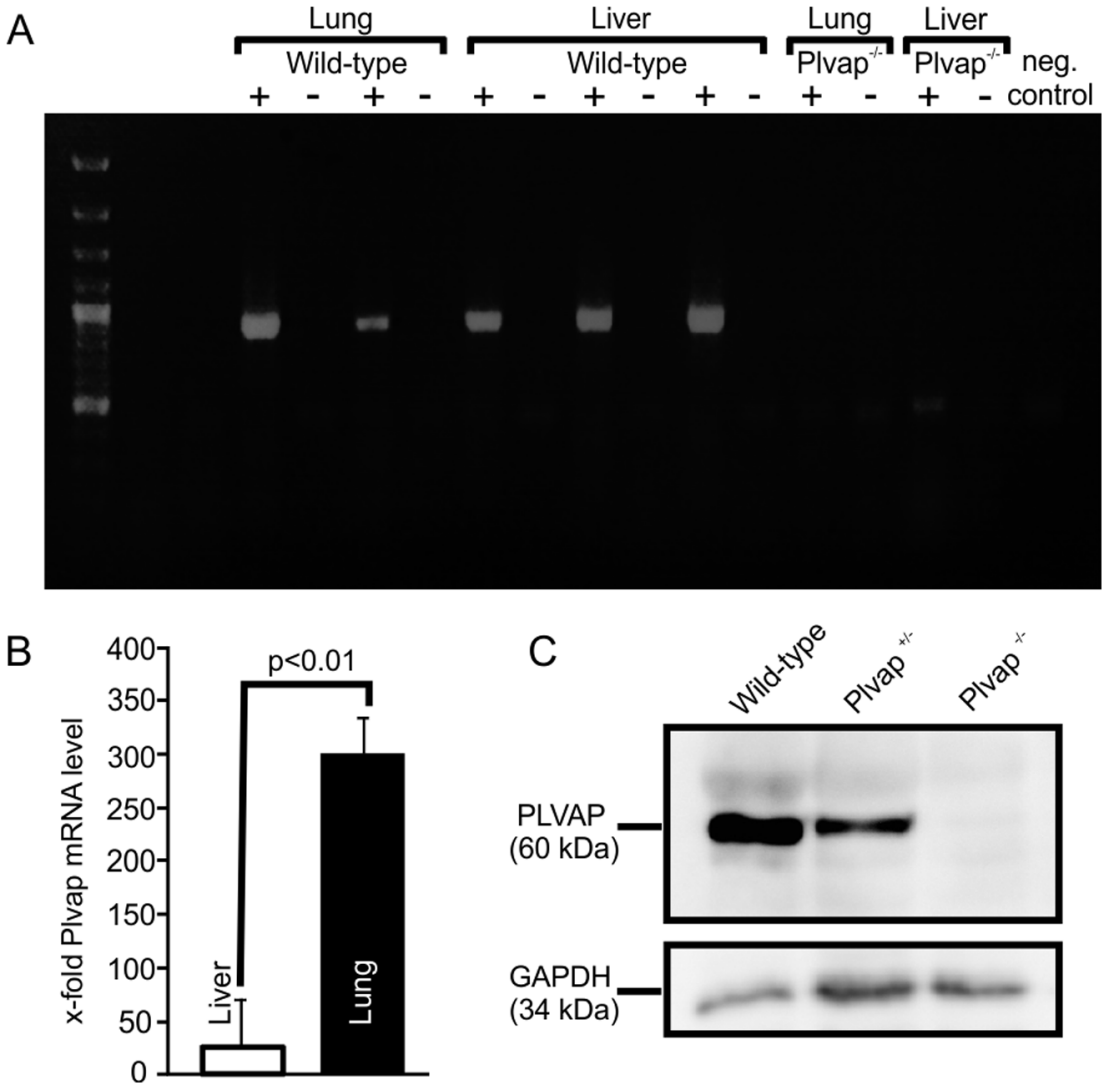
*Plvap* expression in the liver. **A**, RT-PCR analysis from lung and liver tissues. A 901 bp fragment illustrates the mRNA expression of *Plvap* in the liver (+). cDNA from lung tissues served as positive control. RNA that was not reverse transcribed into cDNA (−) and a non-template reaction served as negative controls. No specific band was amplified from cDNA of *Plvap* ^-/-^ lung and liver. **B**, Real-time RT-PCR for *Plvap* with RNA from wild-type liver (n = 4) and lung (n = 3) at P17 to P18. GAPDH was used as a reference gene. **C**, Western blot analysis for PLVAP in proteins isolated from lungs of wild-type, *Plvap^+/-^* and *Plvap^-/-^* animals at P25.

To analyze the expression of *Plvap* mRNA, we performed RT-PCR to amplify a fragment at the predicted size of 901 bp from RNA of wild-type lung and liver, but not in that from *Plvap*-deficient tissues (Fig 2A). When the expression of *Plvap* was analyzed by quantitative real-time RT-PCR in RNA from wild-type tissue at P17 to P18, we observed a 11.5-fold higher mRNA expression level in the lung compared to the liver (Fig 2B), a finding that was consistent with the reported high *Plvap* expression in the lung [13], and the weak *Plvap* promoter activity in mouse liver as assessed by β-galactosidase staining in our study. Western blot analysis with proteins isolated from lungs of wild-type, *Plvap^+/-^* and *Plvap^-/-^* animals at P25 confirmed the specificity of the monoclonal antibodies used to detect PLVAP (Fig 2C).

**Figure 3:**
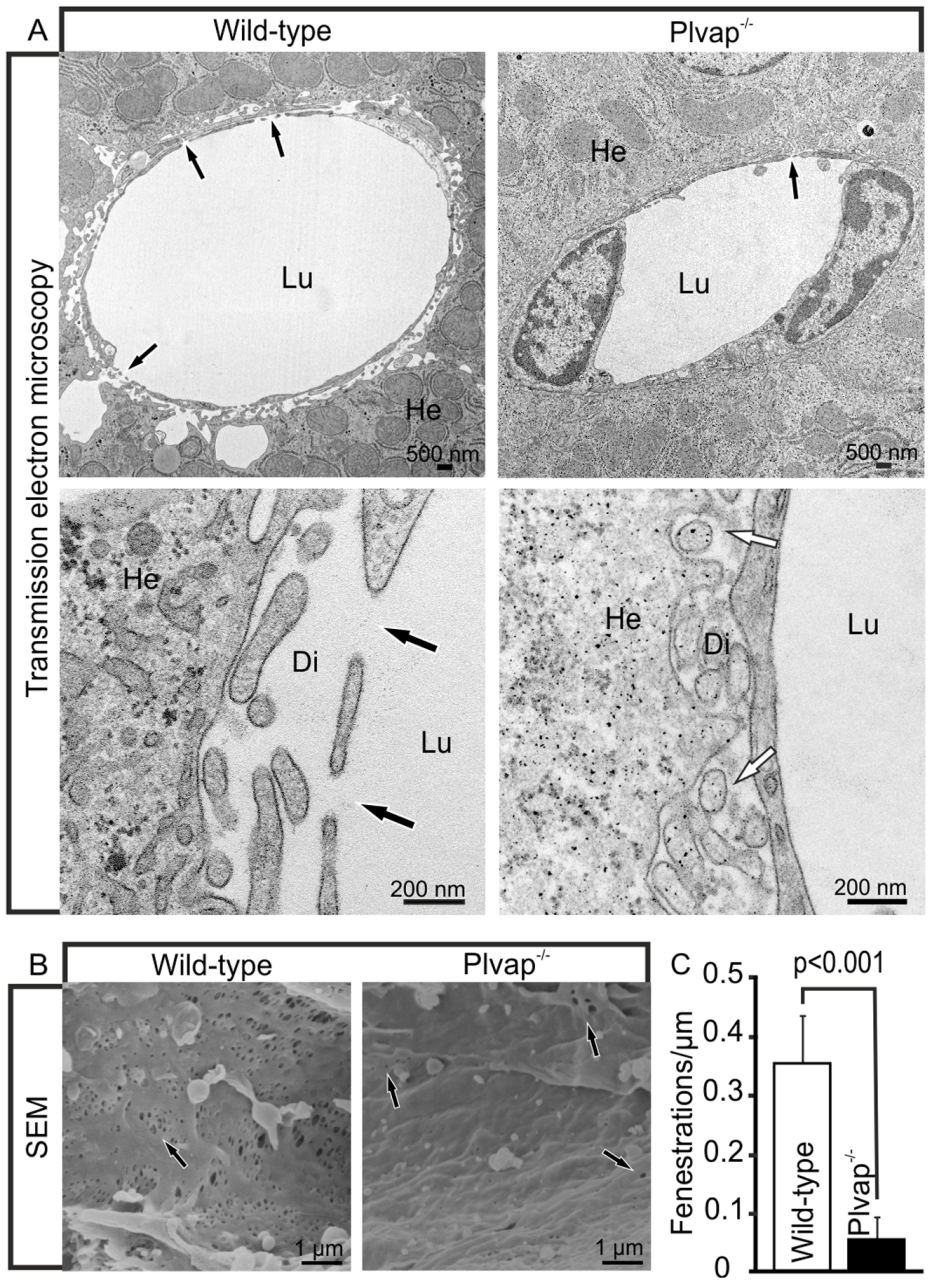
Ultrastructure of *Plvap*-deficient sinusoids. **A**, TEM of a 3-week-old wild-type animal shows numerous fenestrations in sinusoidal endothelial cells (black arrows). In contrast, fenestrations are very rare in the *Plvap* ^-/-^ littermate (black arrow). Lower panels show magnification. While in the wild-type animal Disse's space (Di) is open, it is compressed and filled with fine granular electron-dense material in the *Plvap*-deficient littermate (white arrows). He, hepatocyte, Lu, sinusoidal lumen. **B**, SEM of a 3-week-old wild-type animal shows the arrangement of fenestrations (arrows) in sieve plates. In contrast, sinusoidal endothelial cells of a *Plvap*-deficient littermate show only few fenestrations (arrows) which form individual openings. **C**, Quantitative analysis of fenestrations per µm endothelial length in sinusoids of wild-type and *Plvap* ^-/-^ animals. N = 5, mean ± SEM, ^***^p = 0.00069.

We next analyzed whether sinusoidal endothelial cells of *Plvap*-deficient mice show ultrastructural changes when compared to wild-type littermates. By TEM, numerous fenestrae were observed in sinusoidal endothelial cells of wild-type animals at three weeks of age (Fig. 3A). In contrast, fenestrae were only very rarely observed in the sinusoidal endothelium of *Plvap*-deficient littermates (Fig. 3A). Moreover, in *Plvap^-/-^* mice the space of Disse was compressed and filled with fine granular electron-dense material which was observed adjacent to the hepatocyte microvilli and which was not present in wild-type littermates (Fig. 3A). SEM analyses confirmed the lack of fenestrae in sinusoidal endothelial cells of *Plvap*-deficient mice. Accordingly, sinusoidal endothelial cells in wild-type mice displayed numerous fenestrae that were arranged in sieve plates (Fig. 3B). In contrast, sinusoidal endothelial cells of *Plvap*-deficient mice showed only very few fenestrations which were not arranged in clusters, but rather formed individual openings instead (Fig 3B). The diminished porosity of the sinusoidal endothelium was verified by a quantitative analysis of the number of fenestrations and a highly significant (p≤0.001) and approximately 6.2-fold reduction in the number of fenestrae was observed in *Plvap*-deficient mice (Fig. 3C).

### The lack of fenestrations leads to a decrease in the permeability of sinusoidal endothelial cells

**Figure 4:**
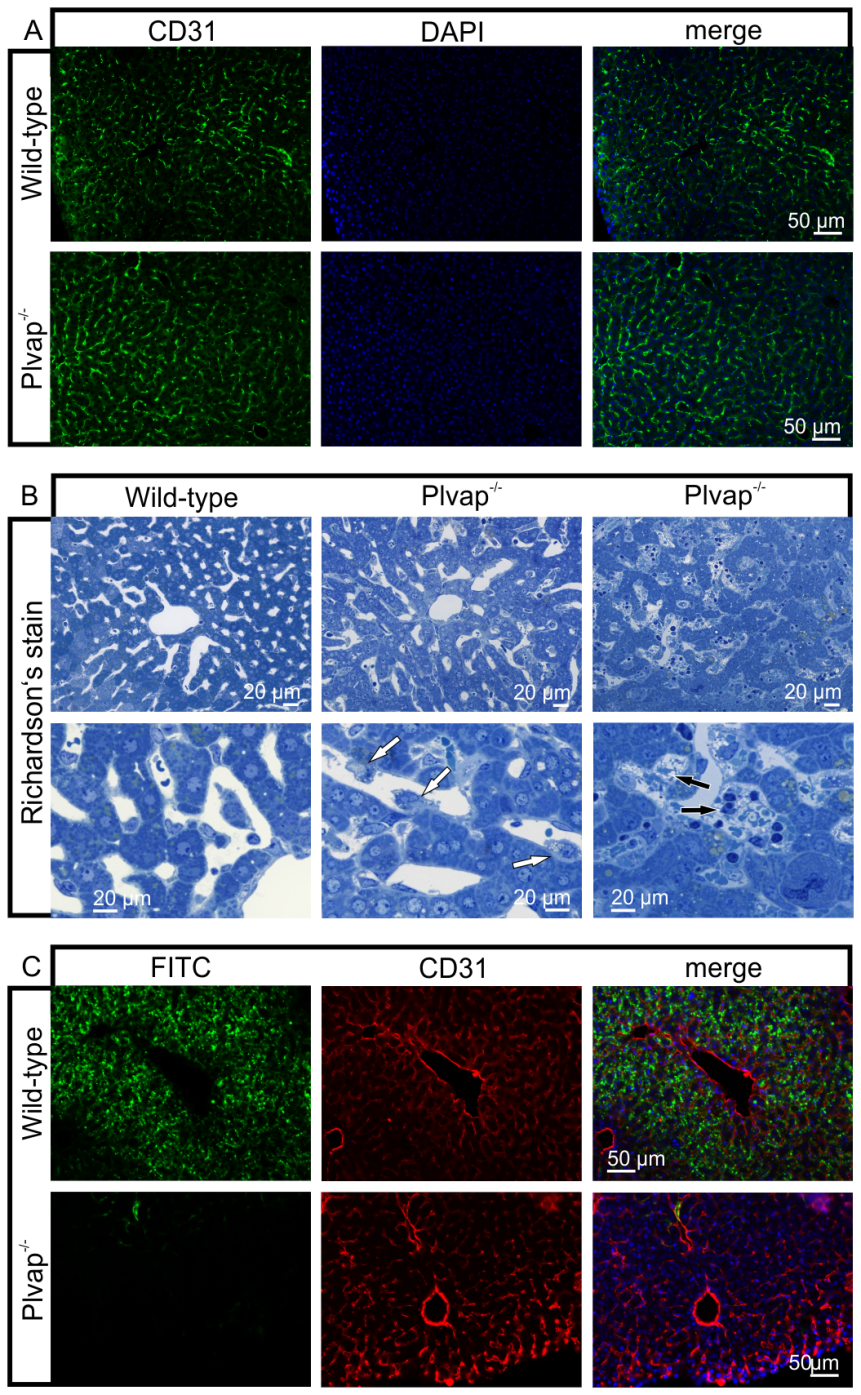
Diminished permeability of liver sinusoids in *Plvap*-deficient mice. Neither by immunohistochemistry with antibodies against CD31 (**A**) nor by light microscopy of 1 µm semi-thin sections (**B**, Richardson's stain) obvious differences are detected with regards to the overall orientation and the density of liver sinusoids between 3-week-old *Plvap* ^-/-^ mice and wild-type littermates. Sinusoids of *Plvap*-deficient mice show a higher number of macrophages in their lumen (white arrows) and focal areas with accumulations of mononuclear cells in Disse's space (black arrows). Lower panels in **B** show higher magnifications. **C**, After perfusion of a wild-type animal with FITC-dextran, a strong FITC-signal (green) throughout the liver is detected. Immunolabeling with CD31 (red) suggests that FITC-dextran molecules have accumulated in the space of Disse. In contrast, in the *Plvap*-deficient littermate, the signal for FITC-dextran is much weaker and barely detectable. Nuclear DNA is labeled with DAPI (blue).

We next analyzed if the absence of PLVAP and the decrease in the number of fenestrations in sinusoidal endothelial cells was associated with major changes in sinusoidal architecture. To this end we labeled the vascular endothelial cells of the liver with antibodies against CD31 and detected no obvious differences between *Plvap^-/-^* mice and wild-type littermates with regards to orientation and density of positively labeled vascular endothelial cells (Fig. 4A). No obvious structural differences in the architecture of liver sinusoids were observed when semi-thin sections were studied (Fig. 4B). A distinct difference though between the sinusoids of *Plvap*-deficient mice and that of wild-type littermates was the higher number of mononuclear cells (presumably Kupffer cells) in the lumen of the *Plvap^-/-^* sinusoids (Fig. 4B). In addition, livers of *Plvap^-/-^* mice showed areas with accumulations of mononuclear cells in Disse's space (Fig. 4B). We next tested the permeability of the sinusoids for macromolecules by perfusion with 70 KDa FITC-dextran possessing a hydrodynamic diameter of approx. 10 nm *via* the portal vein. After perfusion of wild-type animals, a strong FITC-signal was detected throughout the entire liver. Immunolabeling with CD31 strongly suggested that numerous FITC-dextran molecules had passed the endothelial cells via their fenestrae and had accumulated in the space of Disse (Fig. 4C). In contrast, in *Plvap*-deficient littermates a signal for FITC-dextran was barely detectable (Fig. 4C). In a parallel approach, we used 25 nm diameter quantum dots [28] holding a CdSe-containing core for portal vein perfusion. The core allowed us to measure the amount of cadmium per kg liver tissue by inductively coupled plasma mass spectrometry (ICP-MS), which correlates with the amount of quantum dots in the tissue after perfusion. Only 0.05 mg/kg cadmium was detected in the liver homogenate from a *Plvap*-deficient mouse while the liver of the wild-type littermate contained 1.2 mg/kg cadmium. Overall, our experiments strongly supported the assumption of a constrained passage of macromolecules and nanoparticles through the hepatic sinusoidal endothelium of *Plvap*-deficient mice.

### Lack of sinusoidal fenestrae in *Plvap*-deficient mice causes hyperlipoproteinemia and steatosis

**Figure 5:**
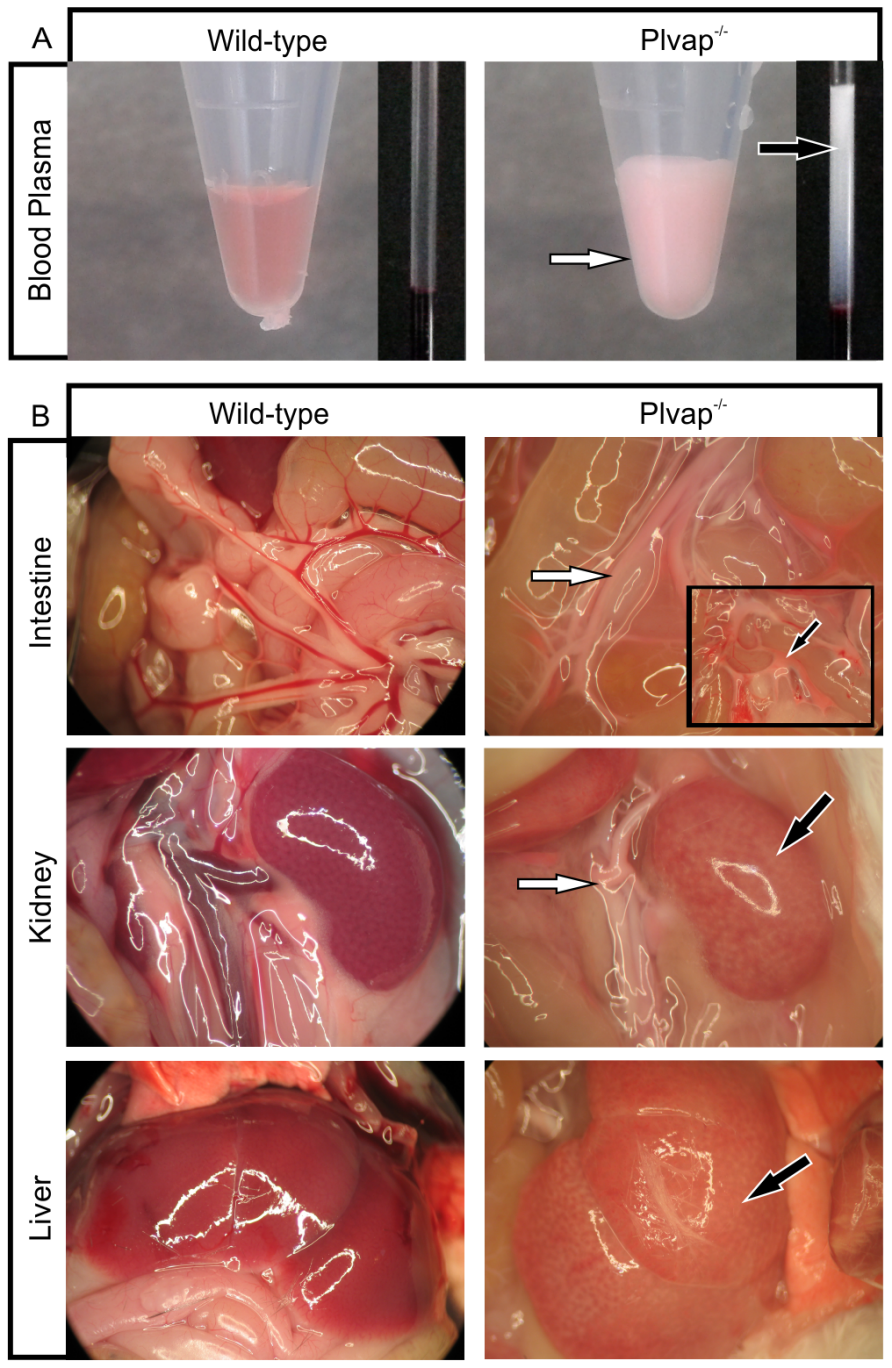
Hyperlipoproteinemia in *Plvap*-deficient mice. **A**, Plasma of a P10 *Plvap*-deficient pup looks milky (arrows), while that from a wild-type littermate is clear. No obvious differences in hematocrit readings are observed between a *Plvap* ^-/-^ pup and its wild-type littermate (right-handed panel). **B**, The blood in the mesenterial or kidney vessels of a 3-week-old *Plvap*-deficient animal is milky (white arrows), while that of wild-type animals is red. Boxed area displays a magnification of mesenteric capillaries that are cut open to show milky blood (black arrow). Kidneys and livers of wild-type animals are of reddish color (black arrows), while those of *Plvap*-deficient littermates are pale.

We next hypothesized that the loss of sinusoidal fenestrations in *Plvap^-/-^* mice should impair the passage of chylomicron remnants to hepatocytes, and analyzed the animals for signs of hyperlipoproteinemia. Plasma isolated from 10-day-old breastfeeding *Plvap*-deficient pups was whitish, milky and thus strongly indicative of hyperlipoproteinemia (Fig. 5A). In contrast, plasma from wild-type littermates appeared clear. Upon macroscopic analysis, the blood in the vessels supplying the small intestine or the kidneys had a milky color, while that of wild-type animals was red (Fig. 5B). In contrast to the kidneys of wild-type animals, which had an intense reddish color, those of *Plvap*-deficient littermates were pale (Fig. 5B). A similar difference in color was observed upon macroscopic analysis of the livers (Fig. 5B). The differences in color were not caused by anemia, as no obvious differences in hematocrit readings were observed between *Plvap^-/-^* pups and their wild-type littermates (Fig. 5A).

**Figure 6:**
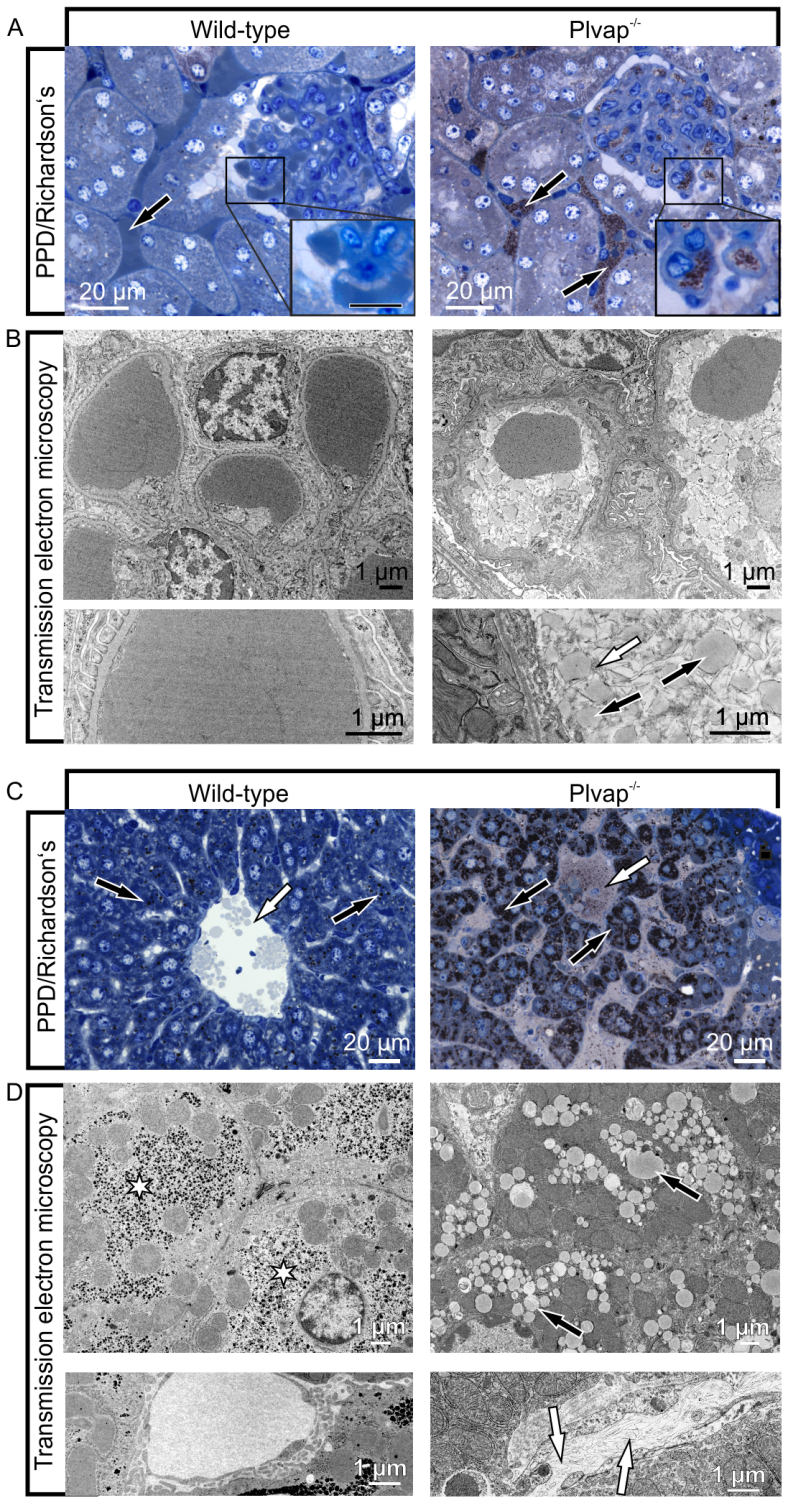
Lipoproteins in capillaries and hepatocytes of *Plvap*-deficient animals. **A**, PPD-labeled granular structures fill the lumen of kidney capillaries in a 3-week-old *Plvap* ^-/-^ mouse (arrows, boxed area, counterstain: Richardson's stain), while only erythrocytes are seen in the wild-type littermate (arrow, boxed area). **B**, The granular structures (black arrows) in the lumen of *Plvap*-deficient capillaries have diameters between 50 to 500 nm and are homogenously electron-dense. The particles are surrounded by extracellular fibrils with the ultrastructural characteristics of fibrin (white arrow) (TEM, lower panel shows higher magnification). **C**, PPD-labeled granular structures are seen in the lumen of liver sinusoids in a *Plvap* ^-/-^ mouse but not in a wild-type littermate (white arrows). The cytoplasm of the vast majority of *Plvap* ^-/-^ hepatocytes is densely filled with PPD-labeled granules (black arrows) which are rare in hepatocytes of wild-type littermates (black arrows). **D**, By TEM, numerous liposomes (black arrows) are seen in the *Plvap*-deficient liver indicating a pronounced microvascular steatosis. Hepatocytes of the wild-type animal contain large aggregates of glycogen (asterisks), which are absent in hepatocytes of the *Plvap*-deficient littermates. The lumen of *Plvap* ^-/-^ liver sinusoids is occluded by fibrillar fibrin (white arrows) suggestive of thrombus formation.

To clarify, if the differences in kidney or liver colors were caused by hyperlipoproteinemia, we analyzed semi-thin sections of mice at three weeks of age, which is close to the maximal life span of our strain of *Plvap*-deficient animals. When kidney sections of *Plvap^-/-^* mice were labeled with the lipid stain PPD, abundant granular structures were identified that filled the peritubular and glomerular capillaries (Fig. 6A). In contrast, in kidneys of wild-type littermates only erythrocytes were seen in the capillary lumen (Fig. 6A). By TEM, the granular structures in the lumen of *Plvap*-deficient capillaries were identified as homogenously electron-dense particles with diameters between 50 to 500 nm (Fig. 6B). The particles were surrounded by extracellular fibrils with the ultrastructural characteristics of fibrin indicating thrombus formation. When semi-thin sections through the livers of *Plvap*-deficient animals were stained, PPD-labeled granular structures were identified in the lumen of liver sinusoids and in Kupffer cells (Fig. 6C). The cytoplasm of the vast majority of hepatocytes was densely filled with PPD-labeled granules (Fig. 6C). Similar granules were only occasionally observed in hepatocytes in wild-type littermates (Fig. 6C). By TEM, the cytoplasmic granules were identified as liposomes indicating a pronounced microvesicular steatosis of *Plvap*-deficient livers (Fig. 6D). Hepatocytes of wild-type animals contained large aggregates of glycogen, which were absent in *Plvap*-deficient littermates (Fig. 6D). Similar to the findings in kidney capillaries, the lumen of liver sinusoids in *Plvap^-/-^* animals was frequently occluded by aggregates of fibrillar fibrin suggestive of thrombus formation (Fig. 6D).

### Increase in plasma chylomicron remnants in *Plvap*-deficient mice

**Figure 7:**
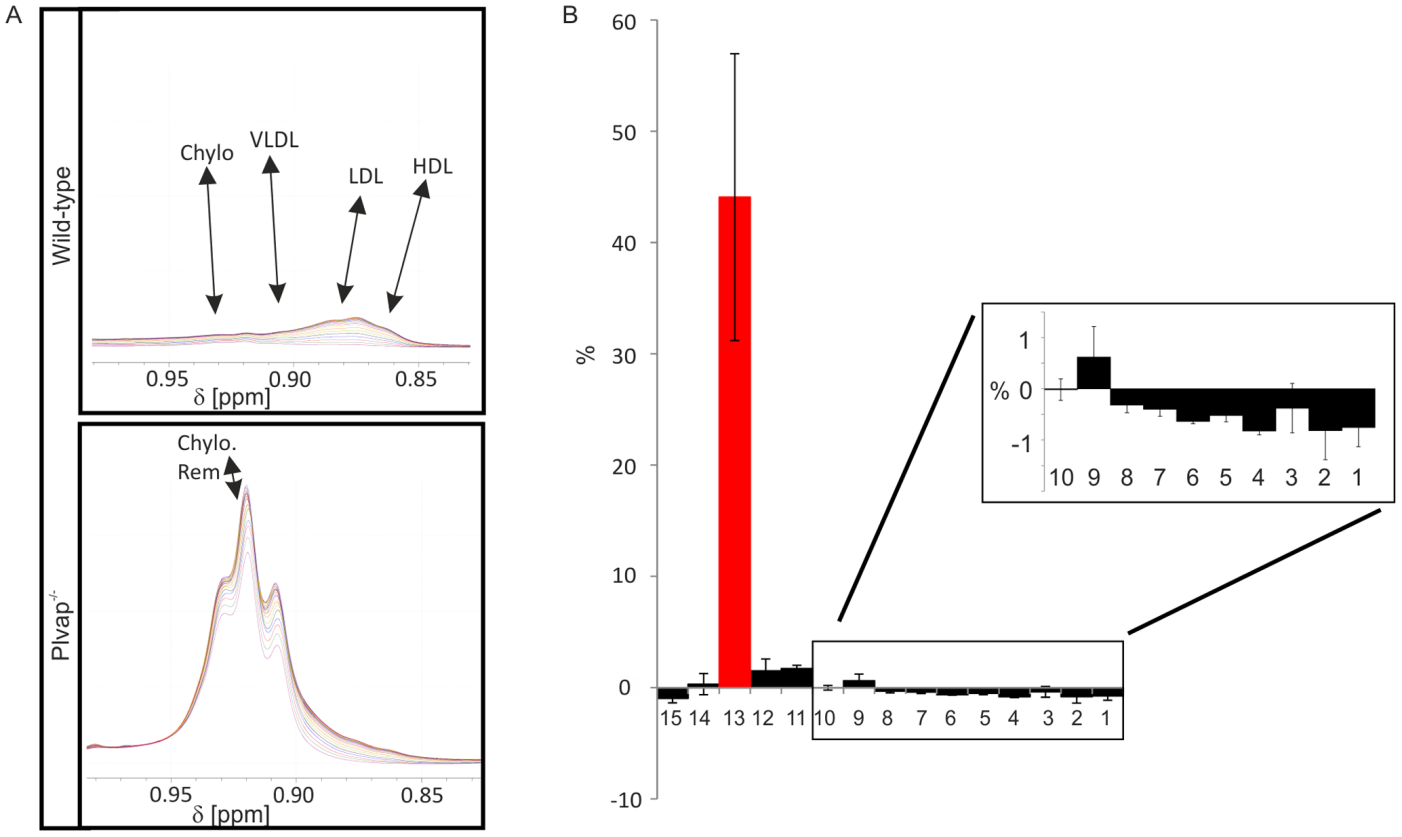
NMR spectroscopy of wild-type and *Plvap*-deficient plasma. **A**, Part of 600 MHz ^1^H NMR spectra of wild-type and *Plvap* ^-/-^ mice at different gradient strengths displaying the (CH3) signals from the methyl groups of the lipids of lipoproteins with maximum gradient strength of 0.535 T/m ±0.01 T/m determined with a water reference sample, the different colors represent different gradient strengths. A strong decrease of the signal intensity corresponds to a small hydrodynamic radius. **B**, Average difference (black bars) of the relative particle numbers ΔN_rel_ in the lipoprotein subclasses L15 to L1^22^ between wild-type and *Plvap* ^-/-^ mice. The red bar indicates the signal intensity difference of the triplet signal visible in the *Plvap* ^-/-^ mice shown in **A**.

To obtain molecular information on the nature of the particles that caused hyperlipoproteinemia in *Plvap*-deficient mice, we performed pulsed gradient stimulated echo NMR experiments and analyzed lipoprotein size and concentration changes between wild-type and *Plvap*-deficient mice (Fig. 7). The data were evaluated analogously to a well-established method for analysis of human lipoproteins by NMR spectroscopy [23], [24]. For the analysis of the nature of the respective lipoproteins, the nomenclature used for human lipoproteins, which are defined by their hydrodynamic radii, was used [23], [24]. Peaks labeled with CH3 containing the signals of the methyl groups of fatty acids of complex lipids and some cholesterol signals were analyzed. Evidently, the shape and intensity of the peaks were different between wild-type and *Plvap^-/-^* mice (Fig. 7A). A triplet signal for lipoproteins with a hydrodynamic radius of 80–100 nm (subclass 13) and largely corresponding to the sizes of chylomicron remnants was increased dramatically in *Plvap*-deficient mice (Fig. 7A, B). A much smaller increase was observed for lipoproteins with sizes between 40 and 80 nm (subclasses 12, 11). Within the limits of error, lipoproteins with sizes between 25 and 40 nm were unchanged, but all smaller lipoproteins (subclasses 8-1) largely corresponding to high-density lipoproteins (HDL) were almost completely abolished in *Plvap^-/-^* mice. The relative concentration of large chylomicrons with diameters >150 nm (subclass 15) was somewhat decreased in the plasma of *Plvap*-deficient mice.

### Steatosis in *Plvap*-deficient mice progresses to necrosis and fibrosis

**Figure 8:**
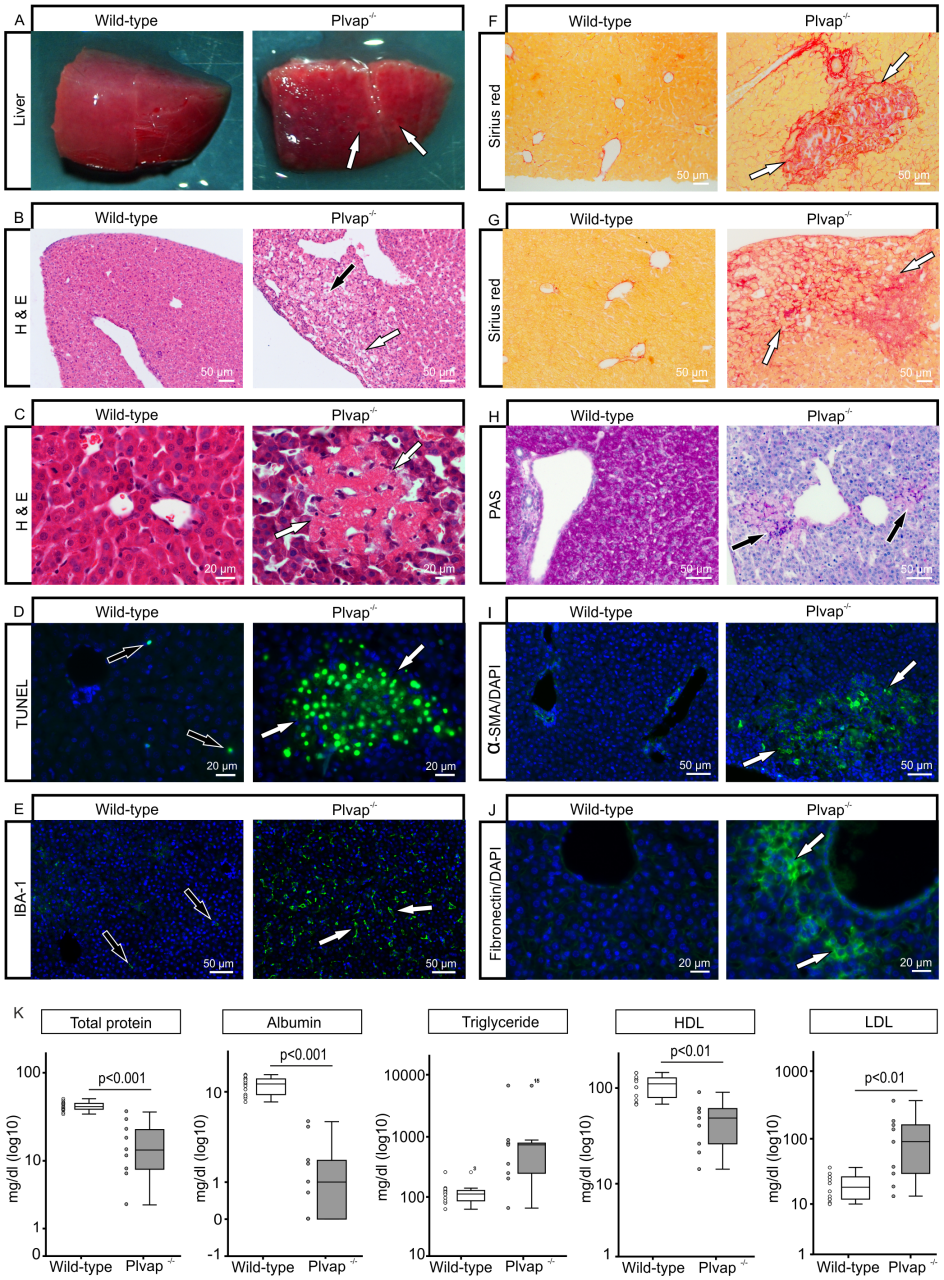
Liver fibrosis and necrosis in *Plvap*-deficient mice. **A**, The surface of a wild-type liver at three weeks of age is smooth while that of a *Plvap* ^-/-^ mouse shows scars (white arrows). **B**, H&E stained paraffin section through the liver of a P17 *Plvap* ^-/-^ animal shows areas with microvesicular and macrovesicular steatosis (white arrow), hepatocyte ballooning (black arrow), and infiltrates of inflammatory cells. **C**, H&E stained paraffin section through the liver of a P18 *Plvap* ^-/-^ animal shows a focal necrotic area (white arrows). Similar areas are not seen in the wild-type littermate. **D**, Numerous TUNEL-positive nuclei (white arrows) accumulate in a focal necrotic area of the P18 *Plvap* ^-/-^ animal shown in **C**. In contrast, only individual TUNEL-positive cells are rarely seen in the wild-type littermate (black arrows). **E**, Immunostaining for IBA-1 (green) at P18 shows a higher number of macrophages (white arrows) in the *Plvap* ^-/-^ liver when compared to the wild-type littermate. **F, G**, Sirius red staining in the liver of an P17 *Plvap* ^-/-^ mouse indicates focal fibrosis (**F**, white arrows) and diffuse fibrosis around sinusoids (**G**, white arrows). In the wild-type littermate, positive staining for sirius red is only seen around larger hepatic vessels. **H**, Intense staining for PAS is seen in the cytoplasm of wild-type hepatocytes. In contrast, hepatocytes are not reactive for PAS in the liver of a *Plvap* ^-/-^ littermate with focal areas of positive extracellular PAS-labeling (black arrows). **I**, α-smooth muscle actin-positive cells accumulate in the liver parenchyma of a *Plvap* ^-/-^ mouse (white arrows), but are only seen in the walls of blood vessels in the wild-type littermate. **J**, A similar area as in **G** shows immunoreactivity for fibronectin in the liver of a *Plvap* ^-/-^ mouse (white arrows). Staining for fibronectin is essentially absent in the liver of the wild-type littermate. Nuclear DNA is labeled with DAPI (blue). **K**. Plasma analysis of wild-type (n = 13) and *Plvap* ^-/-^ mice (n = 9) (mean ± SD).

**Figure 9:**
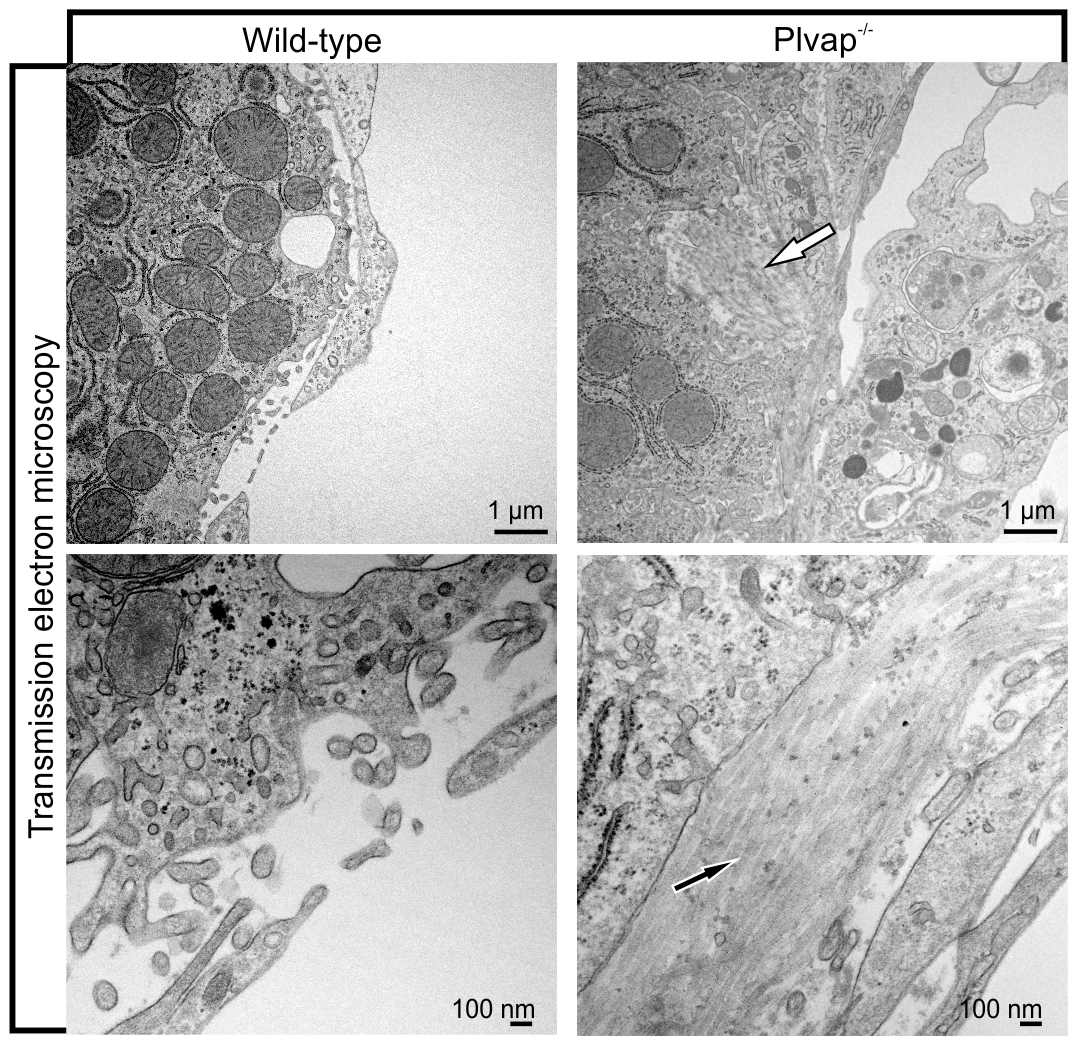
Accumulation of collagen in livers of *Plvap*-deficient mice. By TEM of a *Plvap-*deficient animal at three weeks of age, bundles of collagen fibrils with the typical 67 nm periodicity are seen in Disse's space (arrows). No fibrillar extracellular matrix is present in Disse's space of the wild-type littermate. Lower panels show higher magnification.

Since hepatic steatosis potentially progresses to severe liver disease, we analyzed the livers of three-week-old *Plvap*-deficient mice and observed that the surface of wild-type livers was smooth while that of *Plvap^-/-^* mice showed scar tissue (Fig. 8A). By light microscopy of serially sectioned *Plvap^-/-^* livers at postnatal days (P) 17 to 25, we detected in eight out of eight *Plvap^-/-^* livers that were investigated varying degrees of liver damage. In contrast, damage was consistently absent in livers of the respective wild-type littermates. *Plvap^-/-^* livers presented areas with microvesicular and macrovesicular steatosis, hepatocyte ballooning, and infiltrates of inflammatory cells (Fig. 8B). Moreover, we observed focal necrotic areas (Fig. 8C) containing numerous TUNEL-positive nuclei (Fig. 8D). Immunostaining for IBA-1 at P18 identified a pronounced increase in the number of macrophages in *Plvap^-/-^* livers when compared to wild-type littermates (Fig. 8E). By staining with sirius red, a fine positively-stained network was seen around sinusoids (Fig. 8F, G). In addition, numerous intensely stained focal areas (Fig. 8F) were detected as were areas with more diffuse staining (Fig. 8G), both indicating a pronounced increase in the amounts of extracellular matrix. In contrast, in wild-type littermates positive staining for sirius red was only seen around larger hepatic vessels (Fig. 8F, G). Intense staining for PAS was seen in the cytoplasm of wild-type hepatocytes (Fig. 8H) and correlated with the presence of glycogen aggregates that were identified by TEM (Fig. 6D). Hepatocytes were not reactive for PAS in livers of *Plvap^-/-^* littermates (Fig. 8H). In contrast, numerous focal areas of positive extracellular PAS-labeling were observed (Fig. 8H). By immunohistochemistry of P18 *Plvap^-/-^* livers, we regularly observed foci with accumulations of α-smooth muscle actin-positive cells (Fig. 8I). In the liver, α-smooth muscle actin is a specific marker for reactive hepatic stellate cells (HSC), which are the cellular source of increased extracellular matrix (ECM) formation and deposition in response to hepatic injury [29]. Similar areas showed an increase in immunoreactivity for fibronectin (Fig. 8J). Focal areas that stained for fibronectin or α-smooth muscle positive cells were essentially absent in the livers of wild-type littermates (Fig. 8I, J). Together, these data indicate HSC reactivity and enhanced fibrogenesis in P18 *Plvap^-/-^* livers. To assess functional liver parameters, we performed clinical laboratory tests (Fig. 8K) and observed a significant decrease in the amounts of total protein and albumin in the plasma of *Plvap^-/-^* mice (n = 9) in comparison to that of wild-type littermates (n = 13) (Fig. 8K). In addition, HDL was considerably decreased in plasma samples of *Plvap^-/-^* mice (n = 9) compared to wild-type controls (n = 12) (Fig. 8K). In contrast, the amounts of triglycerides (n = 9) and LDL (n = 9) were increased in the plasma of *Plvap*-deficient animals when compared to that of their wild-type littermates (n = 12 for LDL and n = 13 for triglycerides) (Fig. 8K). In addition, cholesterol was increased more than 3-fold in the plasma of *Plvap^-/-^* mice (380 mg/dl ±119, n = 3) compared to the level of cholesterol in the wild-type controls (103 mg/dl ±30.7, n = 4; p<0.05) consistent with the accumulation of relative cholesterol-rich chylomicron remnants. In some, but not all *Plvap^-/-^* animals, the amounts of AST and ALT were increased (data not shown). When urine of *Plvap^-/-^* animals and wild-type littermates was investigated by SDS-PAGE, no signs of proteinuria were found (data not shown). Finally, we investigated Disse's space by TEM. Here, we regularly observed bundles of collagen fibrils with the typical 67 nm periodicity of collagen types I or III in *Plvap*-deficient animals at three weeks of age (Fig. 9). In contrast, no fibrillar extracellular matrix was seen in Disse's space of wild-type littermates (Fig. 9).

## Discussion

We conclude that PLVAP is required for the formation of fenestrations in liver sinusoidal endothelial cells. The reduced permeability impairs the passage of chylomicron remnants and other lipoproteins such as LDL between the lumen of liver sinusoids and hepatocytes, a scenario that causes hyperlipoproteinemia and results in liver injury. The conclusions rest upon (1) the finding of a substantial reduction in the number of fenestrations (2) the observation of impaired passage of macromolecules and nanoparticles between sinusoids and Disse's space (3) the presence of a massive accumulation of plasma chylomicron remnants (4) the increase in plasma LDL and the reduction in HDL, and finally (5) the development of multivesicular steatosis, steatohepatitis, focal liver necrosis, and fibrosis in *Plvap*-deficient animals.

Our data that show the expression of mRNA for *Plvap* in the mouse liver, albeit at levels that are lower than in organs harboring capillaries with abundant endothelial diaphragms, are essentially comparable to findings of others, which detected *Plvap* mRNA in rat, mouse or human liver [13], [15], [30]. Moreover, similar to our results in the mouse liver, immunoreactivity for PLVAP localizes specifically to sinusoidal endothelial cells in the human liver [31], strongly indicating that its function has been conserved throughout evolution. The important role of PLVAP in transcellular pore formation appears not to be restricted to the formation of fenestrations in the liver. When transcellular pores are generated by vascular endothelial cells to allow leukocyte migration, PLVAP forms a ring that surrounds the migrating lymphocytes and is supported by vimentin filaments which physically interact with PLVAP [32]. Moreover, blocking of PLVAP by antibodies resulted in a decrease in the number of migrating leukocytes [32]. *S*tudies on the mechanism of fenestration formation in liver sinusoids identified cytoskeletal rings around the fenestrations [33], [34]. The formation of fenestrations was significantly induced following experimental disruption of the cortical actin fibers [33]–[35] and originated from a distinct focal area that was termed “fenestrae-forming center” [33]. The entire process occurred rapidly and likely required no *de novo* protein synthesis [33], [34]. It is tempting to speculate that PLVAP molecules in or close to the plasma membrane are required to facilitate local actin disruption and pore formation, processes that are significantly less likely to occur in the absence of PLVAP. PLVAP is essential for the formation of endothelial diaphragms [15], [16] and in recent studies we observed a reduction in the number of diaphragmed fenestrations in the *Plvap^-/-^* pancreas [16] or choroid of the eye [17]. In the liver, we found endothelial diaphragms in wild-type embryonic sinusoids, but not after birth and regard it as unlikely that the formation of an endothelial diaphragm by PLVAP is a prerequisite for the formation of fenestrations in liver sinusoids. Data from *in vitro* studies are consistent with this assumption [33], [34].

The accumulation of chylomicron remnants in the plasma of *Plvap^-/-^* mice supports the concept that the structural characteristics of liver sinusoidal endothelial cells, like the presence of open fenestrations and the absence of a basal lamina, have developed in evolution to allow passage of lipoproteins that are small enough in size [1], [36]. Dietary lipids are absorbed by enterocytes and packaged into triglyceride-rich chylomicrons [37]. Chylomicrons have diameters between 75 nm and 1200 nm [23], [38] and cannot pass the endothelial wall of the diaphragmed fenestrated capillaries in the intestine which allow passage of particles with sizes between 6 and 12 nm [39]. Instead, chylomicrons enter the lymphatic capillaries which have a discontinuous endothelial layer [40]. Chylomicrons reach the blood via the thoracic duct where most of their triglycerides are rapidly removed by lipoprotein lipase (LPL), an enzyme localized at the surfaces of capillaries in adipose and muscular tissues. Cholesterol-rich chylomicron remnants are finally taken up in the liver, a process that requires passage across the sinusoids and binding to both LDL receptor (LDLR) and LDL receptor-related protein (LRP) on hepatocytes [41], [42]. Mice that are deficient in LDLR and LRP show a comparable plasma increase in the amounts of chylomicron remnants and total cholesterol as *Plvap^-/-^* mice [42].

It is tempting to speculate that the reduced number of fenestrations in *Plvap^-/-^* mice hinders not only the passage of chylomicron remnants, but also that of hepatocyte-derived lipoproteins towards the sinusoidal lumen. Our methods do not allow to specifically assess the plasma amounts of very low density lipoproteins (VLDL), since the hydrodynamic radii of chylomicron remnants and VLDL overlap, and chylomicron remnants trapped in the plasma are further reduced in their diameters by the activity of LPL. Still, the increase in the plasma amounts of LDL and the markedly reduced amounts of HDL indicate that lack of fenestrations in sinusoidal endothelia does not only interfere with the passage of lipoproteins from the sinusoidal lumen to Disse's space, but also *vice versa*. VLDL are assembled in hepatocytes and converted to LDL in the plasma, while HDL are formed at the surface of hepatocytes [43], [44]. Liver-derived lipoproteins which are not capable of passing the endothelial layer of *Plvap^-/-^* sinusoids would not accumulate in Disse's space, but be drained by the liver lymphatics to the abdominal lymph nodes [45]. A reduced protein synthesis in *Plvap^-/-^* hepatocytes compromised by steatosis and steatohepatitis will affect lipoprotein synthesis and provides an alternative explanation for the reduced plasma amounts of HDL. In support of this possibility are the reduced plasma amounts of total protein and albumin in *Plvap^-/-^* animals. Finally, the loss of endothelial diaphragms in capillaries outside the liver might be a contributing factor to hypoproteinemia and the low plasma levels of small size lipoproteins such as HDL. The plasma proteins might increasingly pass through fenestrations that are normally bridged by a diaphragm, but are more permeable in *Plvap^-/-^* mice.

Stan and colleagues reported recently on the phenotype of *Plvap*-deficient mice that were bred in a genetic background (hybrid intercrosses containing a mix of Balb/c, C57Bl/6J and 129Sv/J) different from our animals [15]. The authors observed similar plasma changes as reported here. Accordingly, *Plvap^-/-^* (Balb/c, C57Bl/6J, 129Sv/J) mice showed a comparable increase in the plasma amounts of larger lipoproteins and cholesterol, and a decrease in HDL, total protein and albumin. The authors argued that the increase in larger lipoproteins might be a sequel to hypoproteinemia, which may cause depletion of endothelial LPL in animals [46]. We regard this possibility as unlikely, as our data clearly show a specific increase in the amounts of chylomicron remnants, while the amounts of chylomicrons were unchanged or decreased in the plasma of *Plvap^-/-^* mice. In support of our assumption are data which show a correlation between experimental loss of fenestrations and the impaired passage of small chylomicrons in rat livers treated with poloxamer 407, a synthetic surfactant [47]. We suggest that an analysis of the number of fenestrations and their permeability to macromolecules in the sinusoids of *Plvap^-/-^* (Balb/c, C57Bl/6J, 129Sv/J) mice might show changes comparable to that seen in our study.

For the synthesis of VLDL, hepatocytes require triglycerides, which derive from chylomicron remnants, from *de novo* lipogenesis or from albumin-bound fatty acids [48]. It seems reasonable to assume that the reduced availability of chylomicron remnants stimulates *Plvap^-/-^* hepatocytes to increase fatty acid synthesis from carbohydrates. Our observation of a decrease in the amounts of hepatocyte glycogen supports this assumption. A chronic increase in lipogenesis is a contributing factor for non-alcoholic fatty liver disease [49] and may well cause or contribute to steatosis in *Plvap^-/-^* mice. The release of proinflammatory signals from reactive Kupffer cells that ingested chylomicron remnants trapped in the sinusoidal lumen are likely to induce steatohepatitis. Consequently, hepatocytes of *Plvap^-/-^* mice might die by apoptosis while reactive HSC transdifferentiate to myofibroblasts, a key event in the pathogenesis of liver fibrosis [50], [51]. In general, the number of sinusoidal fenestrations decreases significantly in liver fibrosis [50], [52], a process that occurs in parallel with the formation of a continuous basal lamina and has been termed “capillarization” [53]. Sinusoidal capillarization may precede liver fibrosis, but it is unclear if it has the potential to initiate, directly or indirectly, fibrosis [51], [54] Our results appear to answer this question since in *Plvap^-/-^* mice the targeted null-mutation of an endothelial-specific molecule primarily induces the lack of sinusoidal fenestrations and only secondarily, directly or indirectly, leads to liver fibrosis.
